# Effect of Gold Nanoparticle Size on Their Properties as Contrast Agents for Computed Tomography

**DOI:** 10.1038/s41598-019-50332-8

**Published:** 2019-10-17

**Authors:** Yuxi C. Dong, Maryam Hajfathalian, Portia S. N. Maidment, Jessica C. Hsu, Pratap C. Naha, Salim Si-Mohamed, Marine Breuilly, Johoon Kim, Peter Chhour, Philippe Douek, Harold I. Litt, David P. Cormode

**Affiliations:** 1 Department of Radiology, University of Pennsylvania, 3400 Spruce St, 1 Silverstein, Philadelphia, PA 19104 USA; 2 Department of Bioengineering, University of Pennsylvania, 3400 Spruce St, 1 Silverstein, Philadelphia, PA 19104 USA; 3 Department of Medicine, Division of Cardiovascular Medicine, University of Pennsylvania, 3400 Spruce St, 1 Silverstein, Philadelphia, PA 19104 USA; 4 Department of Radiology, Hôpital Cardio-Vasculaire et Pneumologique Louis Pradel, Lyon, France; 5 Centre de Recherche en Acquisition et Traitement de l’Image pour la Santé (CREATIS), UMR CNRS 5220, Inserm U1044, University Lyon1 Claude Bernard, Lyon, France

**Keywords:** Biotechnology, Engineering, Nanoscience and technology

## Abstract

Computed tomography (CT) is one of the most commonly used clinical imaging modalities. There have recently been many reports of novel contrast agents for CT imaging. In particular, the development of gold nanoparticles (AuNP) as CT contrast agents is a topic of intense interest. AuNP have favorable characteristics for this application such as high payloads of contrast generating material, strong X-ray attenuation, excellent biocompatibility, tailorable surface chemistry, and tunable sizes and shapes. However, there have been conflicting reports on the role of AuNP size on their contrast generation for CT. We therefore sought to extensively investigate the AuNP size-CT contrast relationship. In order to do this, we synthesized AuNP with sizes ranging from 4 to 152 nm and capped them with 5 kDa m-PEG. The contrast generation of AuNP of different sizes was investigated with three clinical CT, a spectral photon counting CT (SPCCT) and two micro CT systems. X-ray attenuation was quantified as attenuation rate in Hounsfield units per unit concentration (HU/mM). No statistically significant difference in CT contrast generation was found among different AuNP sizes via phantom imaging with any of the systems tested. Furthermore, *in vivo* imaging was performed in mice to provide insight into the effect of AuNP size on animal biodistribution at CT dose levels, which has not previously been explored. Both *in vivo* imaging and *ex vivo* analysis with inductively coupled plasma optical emission spectroscopy (ICP-OES) indicated that AuNP that are 15 nm or smaller have long blood circulation times, while larger AuNP accumulated in the liver and spleen more rapidly. Therefore, while we observed no AuNP size effect on CT contrast generation, there is a significant effect of size on AuNP diagnostic utility.

## Introduction

Computed tomography (CT) is a widely used clinical imaging technique that employs an X-ray source and a detector array to form images^1^. CT has the capability of producing images with high spatial and temporal resolution^2,3^. As a non-invasive diagnostic tool, CT provides 3-dimensional anatomical information of specific tissues and organs^3–5^, such as the gastrointestinal tract, cardiovascular system, liver and lung^6^. However, when compared to other clinical imaging modalities, such as nuclear imaging and MRI, CT’s lack of sensitivity towards contrast agents is a major drawback^7^. Nevertheless, CT has been rapidly evolving with new innovations such as multi-energy systems, techniques that use lower radiation doses or have reduced scanning times, and the development of advanced image reconstruction algorithms that have resulted in improved image quality^4^, and so the use of CT remains highly important in both clinical and research settings. Therefore, biomedical researchers are investing significant efforts in developing novel contrast agents for CT imaging^8–10^.

Currently, the primary Food and Drug Administration (FDA)-approved intravenous contrast agents for CT imaging are iodinated small molecules such as iopamidol or iodixanol^11^. However, increasing numbers of adverse events caused by iodine-containing contrast agents are reported every year^12^. The primary side effects of using iodinated agents are allergic reactions and contra-indications from use in patients with renal insufficiency^13^. Moreover, these FDA-approved iodinated contrast agents clear very rapidly from the blood and are not specific for any pathology. Due to the drawbacks of iodinated contrast agents, there has been emerging interest in developing alternative contrast agents for CT imaging and nanoparticle-based formulations are one of the primary foci^8,14^. Compared to small molecule contrast agents, nanoparticle contrast agents have several advantages. In general, nanoparticles can carry higher payloads of contrast generating materials^15^, and their shape, size and surface chemistry are usually tunable for specific biomedical applications^16^. They can have blood circulation times of several hours, while the blood circulation time of iodinated small molecules is measured in minutes^17,18^. AuNP have been extensively proposed as CT imaging contrast agents, as well as for other biomedical applications^10,19^. Due to gold’s high atomic number of 79 and its high density, AuNP possess favorable X-ray attenuation properties^20,21^. In addition, AuNP are inert, biocompatible, and their surface characteristics can be easily modified with different capping ligands^22,23^. Also, relatively mature synthetic techniques have been developed that allow for control over the size and shape of AuNP. These characteristics have encouraged AuNP use in biomedical imaging, especially for CT imaging.

The size of AuNP can influence their optical imaging properties, biocompatibility and utility for therapeutic applications^24^. Some researchers have reported that the size, shape, and concentration of AuNP can influence cell uptake in cell tracking applications^2,19,25^. However, conflicting findings have been reported with regards the effect of AuNP size on X-ray contrast generation. Xu *et al*. and Khademi *et al*. studied AuNP smaller than 60 nm and reported that smaller AuNP have greater X-ray attenuation than larger ones^26,27^. Dou *et al*. investigated effect of AuNP size varying from 3.9 nm to 41.1 nm on CT contrast and they found larger AuNP generated greater contrast than smaller AuNP^28^. However, Ross *et al*. found that, for AuNP with sizes up to 76 nm, X-ray attenuation was independent of particle diameter^29^. Moreover, the amount of attenuating material for a given mass concentration will not change between different nanoparticle sizes, thus should not cause differences in attenuation.

The production of attenuation of an X-ray contrast agent is only one of several metrics of its performance. Its pharmacokinetics and biodistribution are key to disease diagnosis and for selecting the correct scanning protocol. Several studies have indicated that the size, surface charge, and coating of AuNP could strongly affect their biodistribution^29–32^. However, none of the previous studies on this topic used AuNP doses in the 100 s of mg/kg range, i.e. at doses relevant for an intravenous CT contrast agent^33^. Previous studies used doses that were much lower, for example 0.85 mg/kg^34^.

Therefore, to thoroughly understand the effect of AuNP size on CT contrast generation and pharmacokinetics/biodistribution, we report the characteristics of six AuNP formulations that range in size from 4 to 152 nm. Their contrast generation was assessed using three clinical CT scanners, using a range of energies. In addition, scans were done with two microCT systems and an SPCCT system. SPCCT is an emerging CT modality that employs photon-counting CT technology to enable advanced material characterization tasks. It can decompose multiple basis materials from multiple energy bins allowing multi-agent imaging simultaneously^35–38^. To the best of our knowledge, no study has been done on the effect of AuNP size on CT attenuation using a SPCCT system. To ensure the accuracy of our experiments, a range of concentrations were used for each formulation, samples were prepared in triplicate, three slices were measured for each sample and images were read by two readers. The samples were gelled to prevent the nanoparticles from settling. Moreover, we performed *in vivo* CT imaging with these AuNP to observe the pharmacokinetics and biodistribution of PEG-modified AuNP after intravenous administration in mice.

## Methods

### Materials

Gold (III) chloride trihydrate (99.9%), sodium citrate dihydrate, hydroquinone (99.9%), sodium borohydride powder (98.0%) were purchased from Sigma-Aldrich (St.Louis, MI). A clinically-approved iodinated contrast agent, Iopamidol, was obtained from Bracco Diagnostics (Monroe, NJ). 5 kDa m-PEG-thiol was purchased from Creative PEGWorks (Winston Salem, NC, USA). C57BL/6 J mice were obtained from The Jackson Laboratory (Bar Harbor, ME).

### AuNP syntheses

Spherical AuNP ranging from 4 to 152 nm were synthesized. 4 nm AuNP were synthesized using a modified Turkevich method by reducing gold chloride with sodium borohydride^39,40^. 15 nm AuNP were synthesized using the Turkevich method^39^. For the larger AuNP sizes, a seeded growth synthesis method employing gold chloride and hydroquinone was used. This seeded growth method “grew” gold around the 15 nm AuNP seeds, via reduction by hydroquinone. Before the syntheses were performed, aqua regia was used to wash the glassware. Aqua regia was prepared by mixing one part nitric acid and three parts hydrochloric acid. AuNPs of 4 nm were synthesized via a modification of the Turkevich method^39,40^. Briefly, 1.6 mL 1% w/v gold (III) chloride (HAuCl_3_) was dissolved in 100 mL of ultrapure water. 1% w/v sodium borohydride was freshly prepared in cold ultrapure water. HAuCl_3_ was then reduced by addition of 1 mL 1% sodium borohydride solution in a dropwise manner while stirring. After adding sodium borohydride, the color of the mixture changed from pale-yellow to black and then eventually to wine-red. The solution was allowed to stir for 20 minutes before ligand exchange was performed.

AuNP of 15 nm were formed via the Turkevich method^39^. Briefly, 600 μL of 1% w/v HAuCl_3_ was added to 60 mL of deionized water. Shortly after the solution was brought to a boil, 1.8 mL of 1% w/v sodium citrate dihydrate was added. Heating and stirring were continued for the next 10 minutes, before the solution was allowed to cool to room temperature. After adding sodium citrate dihydrate, the color of the mixture changed from pale-yellow to black and then eventually to wine-red. The color transition indicates a successful synthesis. The solution was allowed to stir at room temperature for three hours before ligand exchange was performed.

AuNP ranging from 50 to 152 nm in diameter were synthesized using a seeded growth method which utilizes 15 nm Au seeds as nucleation sites and can be controlled by varying the amount of 15 nm AuNP seeds used^25,41^. The 15 nm AuNP seeds were prepared using the method described above without adding capping ligands. Then, 1 mL of 1% w/v HAuCl_3_ was added to 100 mL deionized water at room temperature. While stirring rapidly, an appropriate volume of 15 nm AuNP seeds were added to solution to create AuNP with desired sizes as provided in Table 1. Greater volumes of 15 nm seeds produced smaller nanoparticles. To initiate the nanoparticle growth reaction, 220 μL of 1% w/v sodium citrate dihydrate was added to the solution, and 1 mL of 0.03 M hydroquinone was added immediately afterwards. A different color transition was observed for each synthesis: the color changed from black to wine-red for 50 nm AuNP, from black to red-brown for 79 nm AuNP, from black to red-orange for 100 nm AuNP and from black to orange for 152 nm AuNP. The solution was allowed to stir for three hours at room temperature before ligand exchange was performed.

**Table 1:** The 15 nm AuNP seeds volume needed for syntheses of AuNP with a range of sizes, the amount of ligand needed to stabilize AuNP, and centrifugation parameters for particle washing.

AuNP size (nm)	Seed volume (μL)	Mass m-PEG (mg)	Centrifugation speed (rcf)	Centrifuge tube
4	N/A	12.61	2500	10 kDa molecular weight cut off (MWCO) tubes
15	N/A	4.20	2500	10 kDa molecular weight cut off (MWCO) tubes
50	3000	2.18	3200	50 mL falcon tubes
79	750	1.41	1450	50 mL falcon tubes
100	300	1.05	750	50 mL falcon tubes
152	100	0.70	350	50 mL falcon tubes

### Ligand exchange

After the synthesis of the AuNP cores, a capping ligand was used to coat particles and provide stability. Ligand exchange with 5 kDa thiolated polyethylene glycol (mPEG-SH) was performed for all AuNP^25,42,43^. For PEGylation of AuNP, an appropriate amount of 5 kDa mPEG was added to each AuNP solution based on the optimal molarity of ligand per surface area of AuNP, as provided in Table 1. The 5 kDa mPEG was dissolved in deionized water at a concentration of 5 mg/mL and added to solution. The solution was allowed to stir for at least 6 hours at room temperature to allow ligand exchange to occur.

After the ligand exchange, all AuNP were concentrated and purified using centrifugation. For 4 and 15 nm AuNP, the particles were first purified three times using 10 kDa molecular weight cut off (MWCO) tubes with deionized water at a centrifugation speed of 2500 rcf for 10 minutes. After purification, 10 mL PBS was added to the same tube and AuNP was concentrated to 1 mL at the same centrifugation speed for another 15 minutes. The concentrated AuNP was then collected. For AuNP of 50 to 152 nm, particles were purified and concentrated with conditions optimized for their sizes. The centrifugation parameters varied depending on the AuNP size, as indicated in Table 1. Briefly, a 50 mL falcon tube containing 50 mL AuNP was centrifuged at a desired speed for 12 minutes. The colorless supernatant was carefully removed, and deionized water was added to the tube to make the total volume of solution 50 mL again. The same procedure was repeated twice to purify the AuNP. Afterwards, the AuNP were collected and transferred into 1.5 mL Eppendorf tubes. PBS was added into tubes making the total volume to be 1.5 mL. Then, the tubes were spun at the same speed as noted in Table 1 for 12 minutes. After centrifugation, the clear solution at the top was discarded, leaving behind the concentrated AuNP product. The concentrated AuNP were then collected. AuNP of all sizes were sterilized using a 0.45 μm syringe filter (EMD Millipore, Billerica MA) and stored at room temperature for further use.

### Transmission electron microscopy

The core size and shape of AuNP were examined with transmission electron microscopy (TEM) using a JEOL 1010 microscope (JEOL USA Inc., Peabody, MA) at 80 kV. 10 μL of diluted AuNP solution was dropped onto carbon-coated copper grids (FCF-200-Cu, Electron Microscopy Sciences, Hatfield, PA). The solution was allowed to evaporate before performing microscopy. The average core size of AuNP was determined by manually measuring particles using ImageJ (National Institutes of Health).

### UV/visible absorption

An Evolution 201 UV-visible spectrophotometer (Thermo Scientific, Waltham, MA) was used to record the UV-vis spectra. In brief, 2 μL of AuNP stock solution was diluted with 1 mL deionized water. The concentration of AuNP stock solutions ranged from around 8 to 15 mg Au per mL, thus the diluted sample ranged from 16 to 30 μg Au per mL. 1 mL of diluted AuNP formulation was added to a cuvette for the measurement.

### Dynamic light scattering and zeta potential

The hydrodynamic diameters and zeta potential of AuNP were measured using a Nano ZA-90 Zetasizer (Malvern Instruments, Worcestershire, UK). In brief, 5 μL of AuNP stock solution was diluted with 1 mL deionized water, and 1 mL of diluted AuNP was added to a cuvette for measurement. The Z-average was reported as the hydrodynamic diameter.

### Inductively coupled plasma optical emission spectroscopy

The concentrations of AuNP solutions were determined by inductively coupled plasma-optical emission spectroscopy (ICP-OES) performed on a Genesis ICP (Spectro Analytical Instruments GmbH, Kleve, Germany). 5 μL and 10 μL of AuNP stock solution were dissolved in 1 mL aqua regia. After 20 minutes, 9 mL deionized water was added to the solution making the final volume of the samples to 10 mL for measurement. The concentration of AuNP was determined by averaging the values from the two samples.

### CT phantom imaging

To assess the CT contrast properties of AuNP of different sizes, they were scanned with both clinical CT scanners and micro CT scanners, as well as a SPCCT scanner. In brief, solutions of AuNP were prepared with concentrations varying from 0 to 20 mg/mL, with each concentration having three replicates. The solutions were then suspended in 1% agarose gel via a 1:1 dilution to result in samples whose concentration ranged from 0–10 mg/ml. Vials containing each AuNP size gel mixture at different concentrations were organized and secured in a plastic rack. In order to prepare the samples for clinical CT phantom imaging, the rack was submerged in a plastic container containing water 21 cm in height to mimic a human abdomen. This CT phantom was imaged using a SOMATOM Force 192-slice clinical CT scanner (Siemens Healthcare, Malvern, PA), a Siemens SOMATOM Definition AS clinical CT scanner, and a GE Revolution clinical CT scanner (GE Healthcare, Waukesha, WI) at 80, 100, 120 and 140 kV. In each case, a matrix size of 512 × 512 with a field of view of 37 × 37 cm was used. The reconstructed slice thickness used for the Siemens SOMATOM Force and GE Revolution was 0.5 mm, while 0.6 mm was used for the Siemens SOMATOM Definition AS (0.5 mm was not an option for this system; 0.6 mm was the closest slice thickness available). A convolution smooth kernel (BR40d) was used for the Siemens SOMATOM Force, and 360 mA X-ray tube currents were used for each voltage in all cases. The acquired images from the three different systems were analyzed using Osirix MD (v.8.0.2 64-bit software). Three slices per tube were selected to measure attenuation values, and a circular ROI was drawn on the coronal view of each tube. The mean attenuation values were recorded in Hounsfield units (HU). The attenuation value at each concentration of each AuNP size was recorded as the average of nine measurements of that concentration. The attenuation rate (HU/mM) was reported for every size of AuNP at each CT energy level.

For micro CT phantom imaging, the same AuNP phantom samples as described above were used. The samples of each AuNP size were organized and secured in separate racks, which were 10 cm long and 4 cm wide. The racks were placed directly on the animal bed. The phantom experiments were carried out using a MILabs U-CT (MILabs, Utrecht, The Netherlands) at 55 kV with slice thickness of 100 μm, in-plane resolution of 100 μm, tube current of 190 μA, and exposure of 75 ms, with an analytical reconstruction kernel applied. The experiment was also carried out using a Molecubes X-CUBE (Molecubes, Gent, Belgium) system at 50 kV, with a slice thickness of 100 μm, an exposure of 75 ms, and a resolution of 100 μm. An analytical reconstruction kernel was also applied. Image analysis was performed using the same methods as described above.

SPCCT phantom images were acquired from a 5 multi-energy bins SPCCT scanner prototype (Philips Healthcare, Haifa, Israel) modified from a clinical CT base. Energy thresholds were set at 30, 51, 62, 72 and 81 keV, with the latter threshold set so as to be close to the K-edge of gold, which is at 80.7 keV^44,45^. Details of the scanner and the imaging methodology have recently been published elsewhere^45–49^. A cylindrical insert made of black polyoxymethylene (POM) 16 cm in diameter with 12 holes 16 mm in diameter was used as the phantom. Tubes with varying concentrations of AuNP suspended in 1% agarose gel were placed in the outer eight holes, as presented in Supporting Fig. 1. Repetitive axial scans were carried out using a tube current of 100 mA and tube voltage of 120 kV^38^. Conventional CT images and material decomposition (MD) images were reconstructed using dedicated calibration and projection based regularized material decomposition^50,51^. MD images are water image, iodine image and K-edge material (Au) images and were obtained as previously reported^45^. The acquired images were analyzed using Osirix MD. Circular ROIs were drawn in the middle of the tube for each AuNP sample of each concentration, and the mean attenuation value or gold concentration in mg/mL was recorded. The attenuation rate (HU/mM) was reported for every size of AuNP.

To minimize image analysis bias, two observers read the CT scans in all cases. Bland-Altman analysis was performed and intraclass correlation coefficients (ICC) were calculated on the attenuation recorded by the two observers for all phantom scans using IBM SPSS Statistics (v25.0, 64-bit) in order to assess the inter-observer variation and reliability.

### Animal experiments

All *in vivo* experimental protocols were conducted with approval from the Institutional Animal Care and Use Committee of the University of Pennsylvania under protocol number 804819, and the National Institutes of Health guide for the care and use of Laboratory animals (NIH Publications No.8023, revised 1978) has been followed. No human participants are involved in the study. *In vivo* imaging experiments were performed using female and male (in a 1:1 ratio) C57BL/6 J mice (n = 6 per group). The mice used were three and a half months old on average. All *in vivo* experiments were performed with AuNP doses of 500 mg Au/kg administered *via* the tail vein. Mice were anesthetized using isoflurane during all imaging experiments.

### CT imaging

All *in vivo* CT imaging experiments were performed using a MILabs U-CT (MILabs, Utrecht, The Netherlands). Mice were scanned prior to injection and at 5, 30, 60, 120 minutes post-injection. CT images were acquired using a tube voltage of 50 kV, tube current of 190 μA, slice thickness of 100 μm, in-plane resolution of 100 μm, exposure of 75 ms, and an analytical reconstruction kernel. The CT images were analyzed by two observers using Osirix MD and the attenuation values in HU were recorded for several organs (i.e. heart, liver, spleen, kidney, thigh) using averaged data from three different slices at each time point. The data is presented as the change in attenuation from pre-injection scans as mean ± SEM. Similarly, to the phantom scan analysis, two readers quantified the CT attenuation for all mice scans. Bland-Altman analysis was performed, and ICC was calculated to data obtained from both observers using IBM SPSS Statistics in order to assess the inter-observer variation and reliability.

### Biodistribution

All mice were sacrificed at 2 hours after injection and blood samples were then collected. The mice were then perfused *via* the left ventricle with 20 mL PBS. After perfusion, several organs (i.e., heart, liver, lungs, spleen, kidney) were collected. The organs were weighed and then minced into small pieces. The small pieces were first digested in 800 μL concentrated nitric acid at 75 °C for 24 hours. Then, 200 μL concentrated hydrochloric acid was added and kept at 75 °C for another 3 hours. 9 mL deionized water was then added to bring the digested samples to 10 mL total volume. Gold content in each sample was measured using ICP-OES as described above. Data is presented as mean ± SD.

### Statistical methods

In all figures, error bars are the standard deviations from the mean. In Table 2, the plus-minus values are standard deviations from the mean measurements of AuNP core diameter and zeta potential. IBM SPSS Statistics software was used for performing Bland-Altman analysis and ICC calculations. Graphpad Prism 7 software was used for all other statistical analysis. For comparing CT attenuation rate from CT phantom scans, a Tukey’s multiple comparison test, with a single pooled variance was used to determine if there is significant difference of attenuation rate between different AuNP sizes. For biodistribution of gold contents in organs, a Tukey’s multiple comparisons test was used to compare the interactions between each AuNP size. To compare the effect of gender on biodistribution, a two-way ANOVA was performed. To assess the consistency of measurements between two observers, the ICC was calculated with a two-way mixed model, as well as a Bland-Altman analysis to analyze the agreement and bias between two observers. For attenuation quantification in *in vivo* CT scans, a Tukey’s multiple comparisons test was carried out to determine the significance level between AuNP sizes.

**Table 2:** Summary of AuNP characterization.

Size (nm)	Core diameter (nm)	Hydrodynamic diameter (nm)	Polydispersity index	Zeta-potential (mV)	Absorbance peak (nm)
4	3.9 ± 1.2	24.1 ± 1.2	0.22 ± 0.03	−6.0 ± 0.3	512
15	14.8 ± 3.9	40.7 ± 0.6	0.26 ± 0.06	−20.3 ± 5.5	521
50	50.6 ± 4.9	69.9 ± 0.1	0.08 ± 0.02	−24.2 ± 0.7	532
79	78.9 ± 10.5	96.9 ± 0.2	0.12 ± 0.01	−32.7 ± 0.3	551
100	99.2 ± 11.3	104.8 ± 0.4	0.17 ± 0.02	−35.5 ± 0.7	575
152	152.3 ± 16.4	140.6 ± 0.6	0.17 ± 0.01	−32.1 ± 1.1	627

The core sizes of the AuNP were calculated from TEM. The hydrodynamic diameters were measured with dynamic light scattering (DLS). The polydispersity indexes were derived from DLS measurements. The plus-minus symbol represents the standard deviation of the measurements in each case.

## Results

### AuNP syntheses and characterization

Spherical AuNPs whose core diameter varied from 4 to 152 nm were synthesized to assess their CT contrast properties (these formulations are referred to by their core diameters throughout this manuscript). mPEG coating was used to modify the AuNP surfaces after the synthesis of AuNP. mPEG is widely used as a capping ligand to coat nanoparticles as it provides high stability, reduces reticuloendothelial system (RES) uptake and increases circulation times versus uncoated nanoparticles^52,53^. These PEGylated AuNP were found to be stable (i.e. no visible aggregation) at room temperature for at least 12 weeks after synthesis.

**Figure 1:**
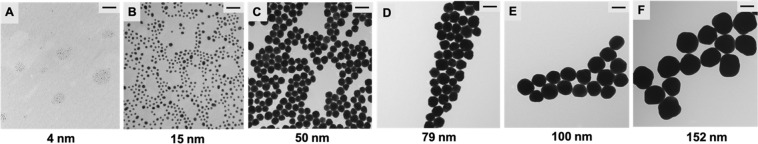
TEM of (**A**) 4 nm, (**B**) 15 nm, (**C**) 50 nm, (**D**) 79 nm, (**E**) 100 nm, (**F**) 152 nm AuNP. The scale bars represent 100 nm in all panels.

TEM was performed to examine the core size and shape of the AuNP (Fig. 1**)**. The AuNP were mostly spherical and of low polydispersity. Core size measurements derived from the micrographs are summarized in Table 2, which indicates success in controlling the AuNP size via our synthetic strategies. The ultraviolet-visible (UV-vis) spectra shown in Supporting Fig. 2 reveals the red shift of peak absorbance as AuNP size increases, as reported by others^25,54^.

We found that the hydrodynamic diameters of the AuNP, as measured by DLS (Table 2), were typically larger than the core diameters as measured from TEM. In general, the Z-average diameters for AuNP are larger than the core size of the particle because hydrodynamic size is a reflection of the AuNP core as well as its protective shell, protective surfactant, hydration layer, as well as other possible stabilizers^55^. However, surprisingly, the diameter from DLS for the AuNP with the 152 nm core was found to be 141 nm. This could be explained by the DLS device’s use of a single refractive index, which can be limiting as the AuNP are composed of a core and a coating that have different refractive indices. The polydispersity indexes (PDI) measured from DLS, shown in Table 2, indicate the low degree of polydispersity though AuNP of all sizes. Moreover, we found the surface charges of the AuNP to all be negative, which was to be expected since they were all stabilized by the same ligand, and were similar to published values^25^.

### CT phantom imaging

#### Clinical CT phantom imaging

**Figure 2:**
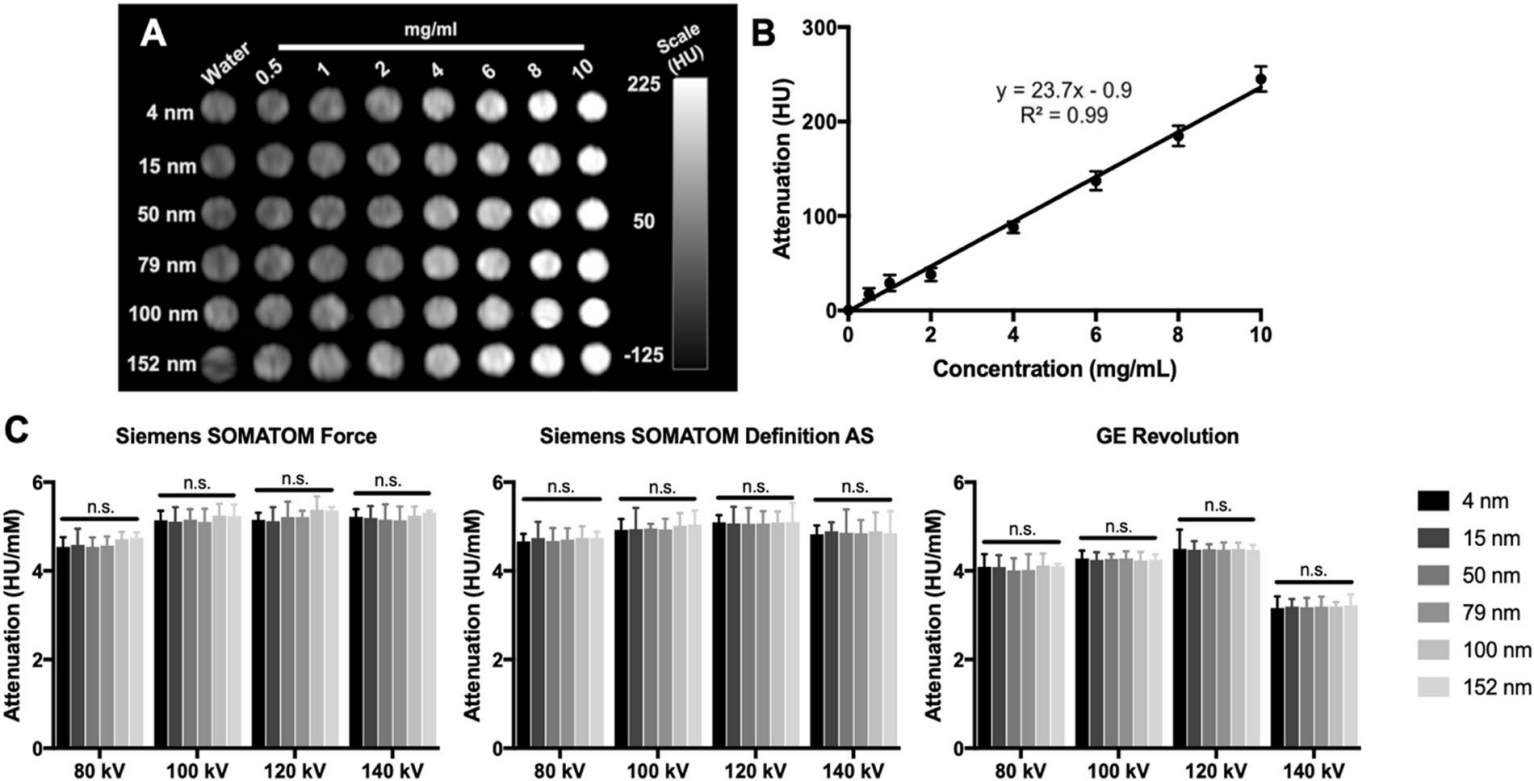
(**A**) Representative sample phantom images from Siemens SOMATOM Force CT scanner. (**B**) X-ray attenuation changes versus concentration for 50 nm AuNP scanned by a Siemens SOMATOM Definition AS CT at 80 kV. (**C**) Attenuation rate values for the AuNP formulation for the scanners noted at 80, 100, 120 and 140 kV.

We performed CT phantom imaging to probe our hypothesis that AuNP size would not affect AuNP CT contrast properties. The generation of CT contrast depends on the concentration of contrast generating atoms in a volume^56^. Therefore, for a given element, no matter what its size, the attenuation should not change at the same concentration level. We investigated the CT contrast properties with AuNP of different sizes using a phantom that contained eight different concentrations of AuNP for each size in triplicate. The samples were surrounded by water 21 cm in height, to simulate the beam hardening experienced in a patient. The phantom was scanned using three different clinical CT scanners: the Siemens SOMATOM Definition AS CT, the Siemens SOMATOM Force CT, and the GE Revolution CT, all with similar scanning parameters. Selected conventional CT phantom images are presented in Fig. 2A. The samples were covered in plastic wrap, which excluded water, therefore isolated tubes are seen in the image. An increase in attenuation from low concentration to high concentration was observed for each AuNP size, as shown in for 50 nm AuNP, for example, in Fig. 2B. The increase of CT attenuation when the mass concentration of AuNP increased was expected^56^. The CT attenuation rates of the different AuNP sizes are presented in Fig. 2C & Supporting Fig. 3. From Fig. 2C it is clear that there is no statistically significant difference in X-ray attenuation between any of the AuNP sizes for any of the three clinical CT systems used.

The phantom results also show that in each case the highest attenuation was observed at 120 kV, consistent with previous reports^57^. This is due to the K-edge of gold being at 80.7 keV, therefore stronger attenuation occurs at scans done at voltages above 80 kV. However, slight differences between attenuation rates were observed among the clinical CT scanners, which are likely due to the differences in the X-ray tubes and patient shielding used for the systems. These results support our hypothesis that AuNP size does not affect X-ray attenuation. In addition, the results of Bland-Altman analysis, as indicated in Supporting Fig. 4, showed that low bias was observed between the two observers. The high ICC value (Supporting Table 1) indicated an excellent agreement of measurement between two observers, further confirming our results.

#### Micro CT phantom imaging

We also performed phantom imaging with two different micro CT systems to further probe our hypothesis that the size of AuNP do not have an impact on X-ray attenuation. We performed phantom imaging studies with a MILabs U-CT and a MoleCubes X-Cube. The data shows, as indicated in Supporting Fig. 3, that there is no statistically significant difference in X-ray attenuation for any of the AuNP sizes. AuNP scanned with the MILabs system had an average attenuation rate of 14.6 HU/mM, as indicated in Supporting Fig. 3, whereas the samples scanned with the Molecubes system had an average attenuation rate of 11.9 HU/mM. The difference in X-ray attenuation rates between the two micro CT systems is likely due to the differences in the X-ray spectra used by the different systems. In general, the X-ray attenuation of AuNP from micro CT was found to be higher than clinical CT. The difference is likely due to the different tube voltages and filtering used for clinical and micro CT, e.g. we used 80 kV to 140 kV for clinical CT, and used 50 kV and 55 kV for micro CT. A different tube voltage can affect contrast generation, depending on the element’s X-ray attenuation characteristics.

The results also support our hypothesis that AuNP size has no influence on X-ray attenuation. In the measurement of CT attenuation in all phantom images, overall interobserver consistency is high. A minimal average bias between the measurements from two observers, as indicated in Supporting Fig. 4A, and the high ICC values (Supporting Table 1), indicate the reliability of the measurement data.

#### SPCCT phantom imaging

**Figure 3:**
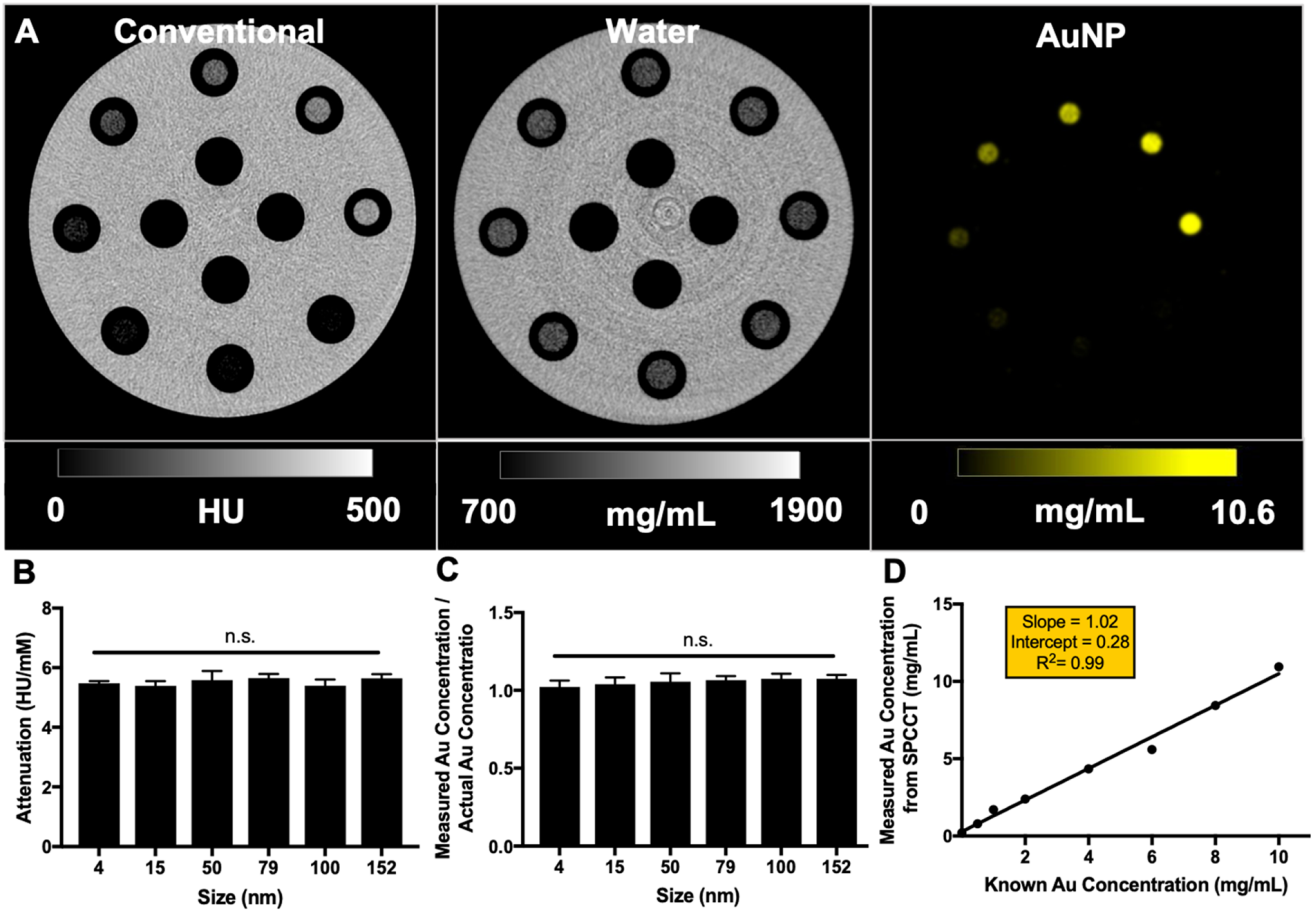
(**A**) Images generated from an SPCCT phantom scan of AuNP of 4 nm ranging from 0 to 10 mg Au/mL in concentration. Conventional CT equivalent image, water image and gold image (left to right). (**B**) Attenuation rate values for the AuNP formulation for the SPCCT scanner at 120 kV. No significant difference of attenuation rate was found between any AuNP formulation. (**C**) Ratios of measured gold concentrations from gold specific images and actual gold concentrations. No significant difference in ratio was found between any AuNP formulation. (**D**) Measured gold concentrations from gold specific images are in agreement with the actual gold concentrations.

We further assessed the contrast generation of AuNP of different sizes using a prototype SPCCT scanner. As expected, gold can be accurately identified in the element specific images (Fig. 3A). Furthermore, no significant difference in contrast generation by AuNP of different sizes was observed, as presented in Fig. 3B and Supporting Fig. 5. The samples scanned by SPCCT have an average of attenuation rate of 5.58 HU/mM, which is comparable to the phantom results from the conventional CT scanners used at 120 kV. In addition, no significant differences in contrast generation were found from gold specific CT scans for AuNP in all sizes – the measured gold concentration from each AuNP size have the same ratio to the known gold concentration, as presented in Fig. 3C. The measured gold concentrations are also in agreement with the actual gold concentrations as shown in Fig. 3D, indicating the accuracy and reliability of the element specific CT scan.

#### *In Vivo* CT imaging

**Figure 4:**
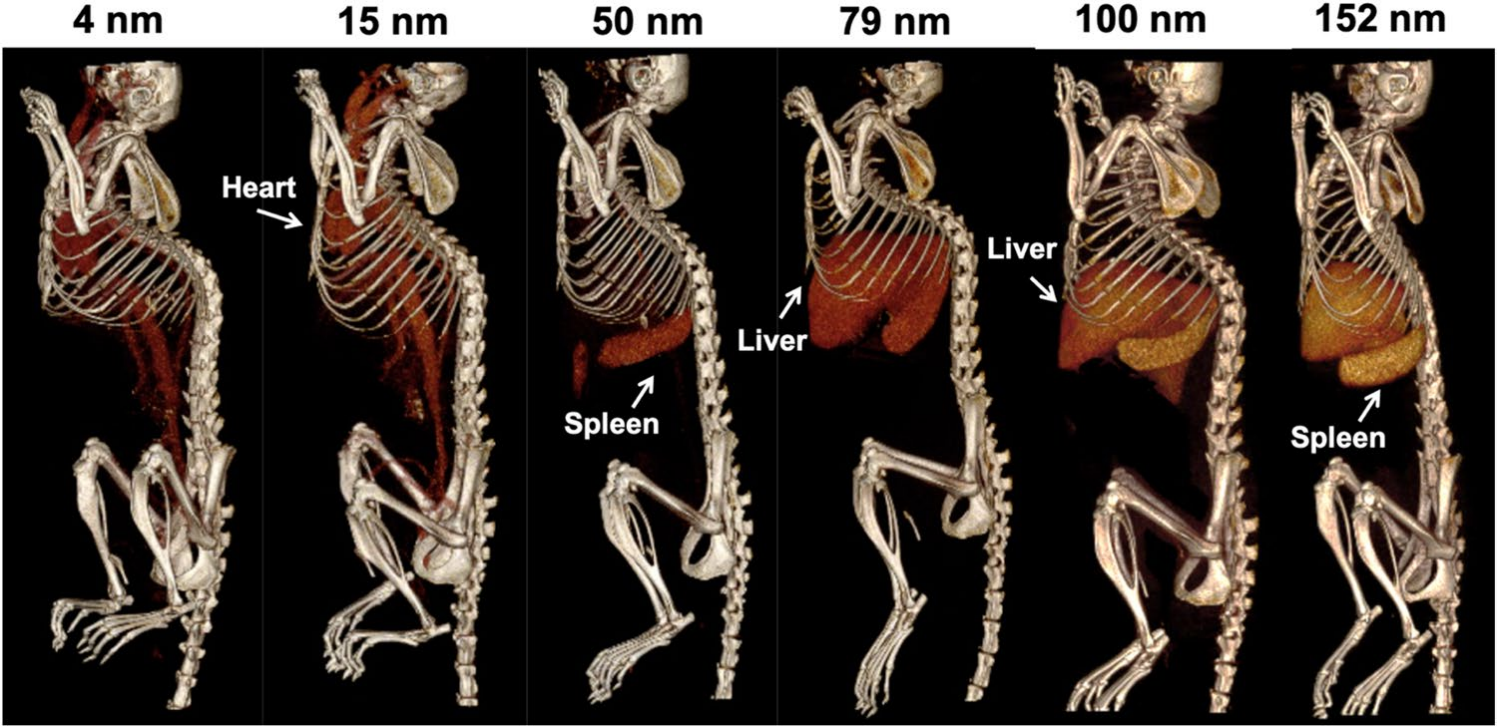
Representative 3D volume rendered CT images at 2 hours post-injection of the AuNP indicated. Images are displayed at a window level of 1090 HU and window width of 930 HU. With this color look up table, the bones of the animal are shown in a bone-like color and contrast arising from the injected AuNP is shown in red to orange-yellow. Images are displayed with a voxel size of 100 μm.

**Figure 5:**
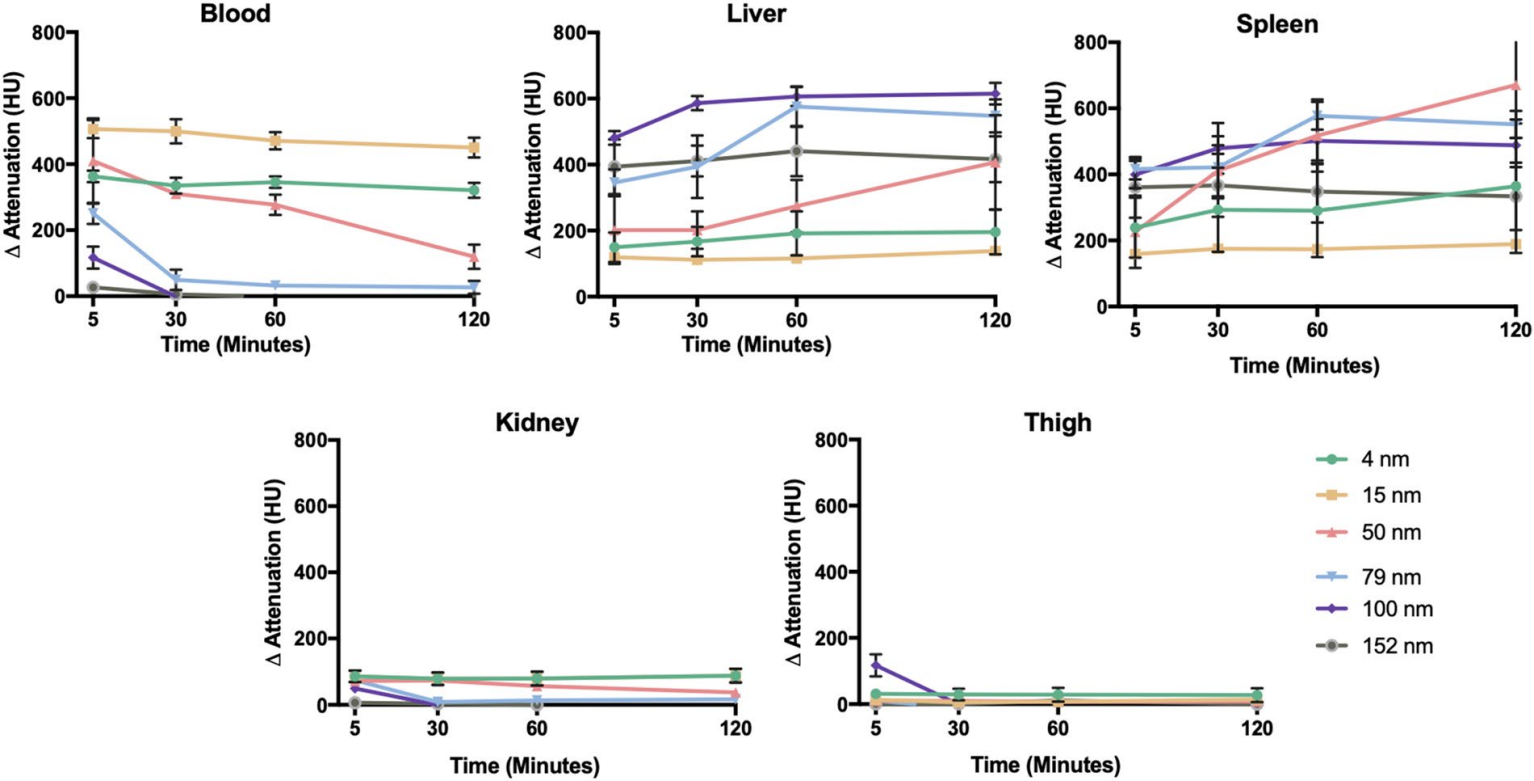
Attenuation changes in different organs over time. A Tukey’s multiple comparisons test was used to compare the changes in attenuation between AuNP sizes. The statistical analysis results are provided in Supporting Table 2.

The pharmacokinetics and biodistribution of AuNP of different sizes were initially assessed via *in vivo* imaging. Mice were pre-scanned, injected intravenously with AuNP of different sizes (500 mg Au/kg) and imaged at various time points with a MILabs U-CT system. The representative CT images acquired two hours after AuNP injection shown in Fig. 4 indicate that strong CT contrast was produced. Images for each timepoint are shown in Supporting Fig. 6. We quantified the contrast in several organs at different time points (Fig. 5). The increase in attenuation in the thigh was close to zero at all time points as expected, due to the tight endothelial junctions in muscle preventing AuNP extravasation. Sustained strong CT contrast was observed in the blood vessels at each time point for 4 and 15 nm AuNP, demonstrating that small AuNP produce strong vascular contrast and have long blood circulation half-lives. On the other hand, minimal contrast was detected in the vasculature at 2 hours post-injection for 50 nm AuNP and larger, indicating more rapid clearance from the blood stream. It is worth noting, however, that for 50, 79 and 100 nm AuNP there was some contrast in the blood at 5 minutes post-injection. Relatively low contrast was observed in liver and spleen for 4 and 15 nm AuNP, whereas contrast gradually increased in the liver and spleen for 50 nm and larger AuNP as time progressed. Low contrast was observed in the kidneys for all AuNP tested and seemed to correlate with the contrast in the blood. Bland-Altman analysis (Supporting Fig. 4B) showed that low bias (i.e. 3.314 HU) was observed between the two observers. A high ICC value (i.e. 0.954) as indicated in Supporting Table 1 further confirmed good agreement between the readers.

#### Biodistribution

**Figure 6:**
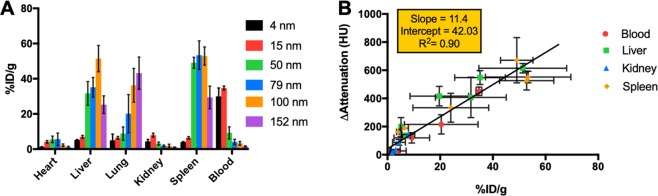
(**A**) Biodistribution of AuNP in different organs at 2 hours post-injection. A Tukey’s multiple comparisons test was done to compare the interactions between each AuNP size. (**B**) Comparison between the attenuation change in different organs derived from CT scans performed at 2 hours post-injection and biodistribution of AuNP in different organs at 2 hours post-injection determined with ICP-OES.

The biodistribution of the AuNP of all sizes at 2 hours post-injection is presented in Fig. 6A. As can be seen, more AuNP were taken up by the lung, liver and spleen for AuNP 50 nm or larger, compared to smaller AuNP. For 4 and 15 nm AuNP, more than 20% of the injected dose (ID) was still present in the blood, indicating reasonably long blood circulation half-lives for these smaller AuNP. No significant differences in gold content were observed for heart for all AuNP sizes at 2 hours post-injection, as shown in Supporting Table 3. The results are in accordance with similar findings of AuNP reported by others that smaller AuNP has longer blood circulation half-life whereas larger AuNP are more easily taken up by liver and spleen^34^.

These biodistribution results are also in agreement with our in *vivo* CT attenuation quantification results as shown in Fig. 6B, with a R-squared value of 0.90 indicating a good correlation between attenuation change at 2 hours post-injection and the biodistribution results.

In addition, we compared the biodistributions of the AuNP between genders for each organ. No significant differences were found between genders for any organ or AuNP size, as presented in Table 3, although small sample sizes were used (n = 3/gender).

**Table 3:** Comparisons of AuNP tissue distribution by mouse gender at 2 hours post-injection.

		4 nm	15 nm	50 nm	79 nm	100 nm	152 nm	P-Value
**%ID/g**								
Heart	Female	1.43	4.46	5.92	7.93	1.17	0.62	0.208
	Male	0.92	3.81	4.95	2.14	3.02	1.50	
Liver	Female	5.03	8.29	27.94	83.77	41.8	30.79	0.062
	Male	9.67	5.69	37.20	41.35	60.93	19.68	
Lung	Female	1.54	6.30	4.18	40.12	50.16	45.82	0.169
	Male	8.38	6.58	15.78	24.17	71.01	52.12	
Kidney	Female	4.31	9.46	4.10	2.37	0.70	1.30	0.217
	Male	4.38	6.39	1.67	0.95	3.02	0.57	
Spleen	Female	4.13	7.87	51.02	61.06	85.74	34.69	0.093
	Male	3.35	5.03	46.14	41.89	49.5	24.04	
Blood	Female	32.30	35.84	12.00	4.45	2.77	1.61	0.180
	Male	8.50	33.61	5.06	3.47	3.63	1.44	

No significant differences were found between gender in each organ.

Significant difference by two-way ANOVA between male and female.

## Discussion

We synthesized PEGylated AuNP within a wide range of gold core diameters (i.e. 4–152 nm) and observed no variation in CT attenuation with AuNP size. Previously, one group reported that X-ray attenuation is independent of AuNP size^29^, whereas two other groups have stated that smaller AuNP provide greater X-ray attenuation than larger ones due to the difference in surface area^26,27^, and another group reported that larger AuNP generate greater contrast than smaller AuNP^28^. However, the amount of attenuating material (gold) will not change between different sizes at the same concentration and so the X-ray attenuation, therefore, should also not change between different AuNP sizes. The difference between our results and other groups’ results could be explained by their methodology. For example, one group investigated concentrations of AuNP in the 0–0.5 mg/mL range, which, as can be seen in Fig. 2B, is effectively below the detection limit of typical CT systems^26^. Furthermore, samples of large AuNP (50 nm and larger) sediment after standing for over 30 minutes and therefore concentration gradients can be created. We used agarose gels to prevent such issues, whereas other groups did not note methodology to prevent such issues^26,27,29^. Their methodology to determine concentrations of AuNP samples was also not made clear, therefore it is possible that errors could arise from that source. Also, previous studies investigating the effect of AuNP size on contrast properties only used one CT system. In our study, we chose to use multiple clinical CT systems from different manufacturers to scan the same phantoms and eliminate the possibility that our results were specific to one type of system - six different CT systems with various manufacturers and applications were employed for phantom imaging experiments to ensure that our findings were generalizable and not specific to an individual system. Thus, we used both clinical CT scanners, small animal micro CT scanners as well as a SPCCT scanner for the phantom imaging experiments to increase the reliability of the experimental results. Use of these CT scanners for the phantom imaging provides clinical relevance to our experiments. The phantom imaging experiments done on both clinical and micro CT indicated that there was no statistically significant difference on X-ray attenuation rate among different AuNP sizes. The experimental results support our hypothesis that AuNP size does not affect X-ray attenuation as the amount of attenuating material does not change with size.

For the purpose of investigating the effect of PEGylated AuNP size on pharmacokinetics and biodistribution, which had not previously been done at CT relevant doses (i.e. 500 mg/kg), *in vivo* imaging experiments were performed using C57BL/6 J mice. As we expected, no adverse effects were observed in mice two hours after intravenously administering AuNP of each size, suggesting that PEGylated 4–152 nm AuNP are safe as contrast agents for CT within the timeframe of this study, however, evaluation of long-term toxicity of these AuNP needs further investigations. PEGylation is one of the features that increase the blood circulation time of AuNP. As expected, and described for many AuNP with coatings of long chain PEG molecules^58,59^, we found a sustained blood circulation for 4 nm and 15 nm AuNP 2 hours after intravenous injection. 50 nm AuNP also persist in the vasculature up to 1 hour after injection, then start clearing from the blood stream. 79 nm and 100 nm AuNP are in the bloodstream at 5 minutes post-injection, but are rapidly cleared from the blood thereafter. 152 nm AuNP were cleared from the blood and taken up in other organs before the 5 minutes post-injection CT scan. Morais *et al*. investigated the effect of surface coating on the biodistribution of 18 nm AuNP and found that majority of citrate coated AuNP were trapped in lung, liver and spleen at 2 hours post injection^60^. Schmid *et al*. studied the biodistribution of AuNP stabilized with sodium 3-(diphenylphosphino)benzene sulfonate (TPPMS) ligands, and they found that AuNP 18 nm rapidly cleared from bloodstream at 1 hour post injection^61^. Compared to those studies using different capping ligands for AuNP, our results showed a prolonged blood circulation time of AuNP of similar size, suggesting the effect of long chain PEG molecules on blood circulation time.

The size of nanoparticle is another major factor that dictates the AuNP pharmacokinetics and biodistribution. As mentioned earlier, we found that 15 nm AuNP and smaller persist longer in the vasculature when compared to 50 nm AuNP and larger, suggesting longer blood circulation half-lives for small AuNP. At the same time, we found that AuNP of 50 nm and larger tend to accumulate in liver and spleen and they rapidly cleared from the bloodstream. Our findings are in agreement with the results from Zhang et.al who investigated PEGylated AuNP of different sizes varying from 20 nm to 80 nm at a dose of 2.6 × 10^11^ AuNP/mouse, and they reported that 40 nm and 80 nm AuNP were cleared from the blood rapidly and were rapidly taken up by liver and kidney, however, 20 nm AuNP had high blood content at all time points^62^. Similarly, the findings from Li *et al*. who also investigated size-dependent biodistribution of PEGylated AuNP from 6.2 nm to 61.2 nm after intravenous injection at a dose of 3 mg/kg, suggested that large AuNP were more rapidly accumulated in liver compared to small AuNP at all time points^63^. Cho *et al*. reported that 100 nm PEGylated AuNP have a shorter blood circulation half-life than AuNP of 4 nm and 13 nm at a dose of 0.85 mg/kg^34^. Our findings confirmed their results, although none of the groups explored the AuNP biodistribution at CT dose levels as high as 500 mg AuNP/kg. It is striking that similar trends in pharmacokinetics were observed in our experiments to those found in previous work, despite using doses that are about 100 times higher. While each formulation between 4 and 100 nm in core diameter provided vascular contrast immediately post-injection, only the 4 and 15 nm AuNP afforded sustained vascular contrast. On the other hand, AuNP in the 50–152 nm range offer effective liver and spleen imaging. However, it is notable that the clearance of larger AuNP we observed in this dose range was much more rapid than that observed with lower doses^62^.

Several other studies on different nanocomposites such as iron oxide nanoparticles^64^, oil-in-water emulsions nanoparticles^65^, and polysulfide nanoparticles^66^, also indicated a decrease in blood circulation half-life with an increase of nanoparticle size. However, the larger nanoparticles in those studies did not clear from the blood as quickly as the large AuNP in this study. The reasons for this difference are unclear, moreover, other nanoparticles of similar size (e.g. liposomes) have long circulation times^67^. This may be due to the fact that liposomes are “soft matter” with flexible structures, whereas AuNP are solid metal^67,68^.

Furthermore, we also compared the biodistribution results between gender for each organ. To the best of our knowledge, no study has reported the effect of gender on biodistribution with AuNP of widely differing sizes. No significant difference was found between gender, although the group sizes that we used were small. Wu *et al*. also investigated the effect of gender on biodistribution after intravenous injection of Au@Ag nanorods in a diameter of 34.6 nm – they found more silver deposition in gland and heart in female rats, and more gold accumulation in male livers after 24-hours post injection^69^. However, this study used different nanoparticle formulations to those herein studied and did not use nanoparticles that ranged in size, and the different nanoparticle shape, administration routes and post-injection time could also have caused the difference between our findings. Nevertheless, biodistribution of AuNP can be attributed to many other physicochemical properties such as surface charge, chemical composition, as well as shape and hardness of nanoparticle as mentioned earlier^70,71^.

Our study has a number of limitations. For example, it is unclear what effect these differing pharmacokinetics have on tumor penetration or accumulation sites of inflammation. Moreover, while we investigated AuNP with core diameters of 4, 15, 50, 79, 100 and 152 nm and found that X-ray attenuation was independent of AuNP size in that range, AuNP with sizes smaller than 4 nm or larger than 152 nm were not studied. All animals were sacrificed after 2 hours injection, so it is unclear how the AuNP are distributed at 24 hours or longer timepoints. Furthermore, we only studied one coating (albeit PEG, the most commonly used nanoparticle coating for biomedical applications) and results can differ depending on the coating used.

## Conclusion

In this study, we have synthesized several AuNP with core sizes ranging from 4 to 152 nm. These AuNP were found to be spherical with low polydispersity. Their X-ray attenuation linearly correlated with AuNP concentration. However, in clinical, micro CT, and SPCCT, X-ray attenuation is independent of AuNP diameter. Moreover, we investigated the effect of AuNP with sizes from 4 to 152 nm on *in vivo* CT contrast at several time points *via* imaging mice and biodistribution after intravenously administrating AuNP. We found that small PEGylated AuNP (i.e., 4 and 15 nm) circulate in the blood longer than larger AuNP (i.e. 50 to 152 nm). On the other hand, we found that larger AuNP provided strong liver and spleen contrast. The difference in pharmacokinetics resulting from size may be important when considering nanoparticle design for diagnostic and therapeutic applications.

## Supplementary information

supplementary Dataset

## Data Availability

The datasets generated during and/or analysed during the current study are available from the corresponding author on reasonable request.
