# Supplementary material for: Effect of Gold Nanoparticle Size on Their Properties as Contrast Agents for Computed Tomography
Source: Sci Rep. 2019 Oct 17;9:14912. doi: 10.1038/s41598-019-50332-8 (PMC6797746; doi:10.1038/s41598-019-50332-8)
Supplement: Supplementary file 1 — supplementary Dataset [file 41598_2019_50332_MOESM1_ESM.pdf]

Effect of Gold Nanoparticle Size on Their Properties as Contrast Agents for Computed Tomography

Yuxi C. Dong[1,2†], Maryam Hajfathalian[1†], Portia S. N. Maidment[1], Jessica C. Hsu[1,2], Pratap C. Naha[1], Salim Si-Mohamed[4,5], Marine Breuilly[5], Johoon Kim[1,2], Peter Chhour[1], Philippe Douek[4,5], Harold I. Litt[1,3], David. P. Cormode[1,2,3] *

Departments of Radiology[1], Bioengineering[2], Medicine, Division of Cardiovascular Medicine[3], University of Pennsylvania, 3400 Spruce St, 1 Silverstein, Philadelphia, PA 19104, USA, Tel: 215-615-4656, Fax: 215-662-7868. David.Cormode@pennmedicine.upenn.edu

Department of Radiology[4], Hôpital Cardio-Vasculaire et Pneumologique Louis Pradel, Lyon, France

Centre de Recherche en Acquisition et Traitement de l'Image pour la Santé (CREATIS)[5], UMR CNRS 5220, Inserm U1044, University Lyon1 Claude Bernard, Lyon, France

* Corresponding Author

† These authors contributed equally.

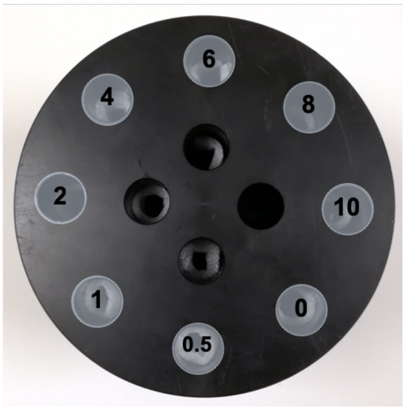

Supporting Figure 1: A phantom setup for the SPCCT scanner. Tubes with the concentrations of AuNP noted (in mg/mL) suspended in 1% agarose gel were placed in the outer eight holes.

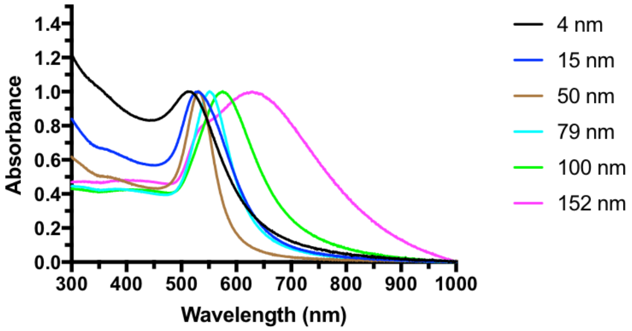

Supporting Figure 2: UV-vis absorption spectra for AuNP size of 4, 15, 50, 79, 100 and 152 nm.

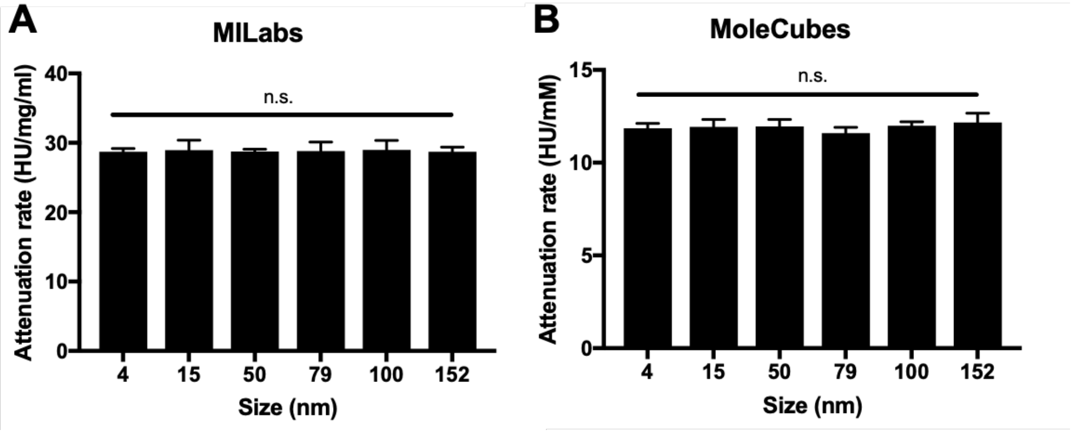

Supporting Figure 3: A) Attenuation rates for the MILabs micro CT system. B) Attenuation rates for the MoleCubes micro CT system.

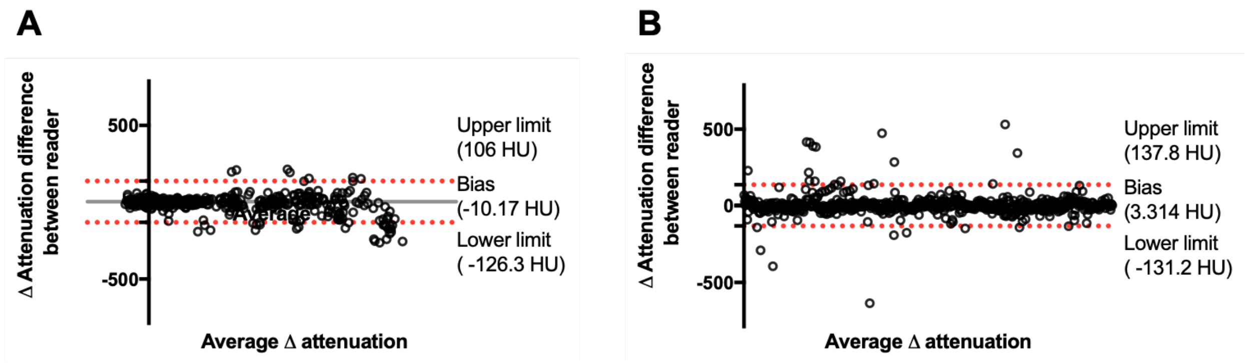

Supporting Figure 4: Bland-Altman plots of the data from the two observers for A) phantom scans and B) *in vivo* scans.

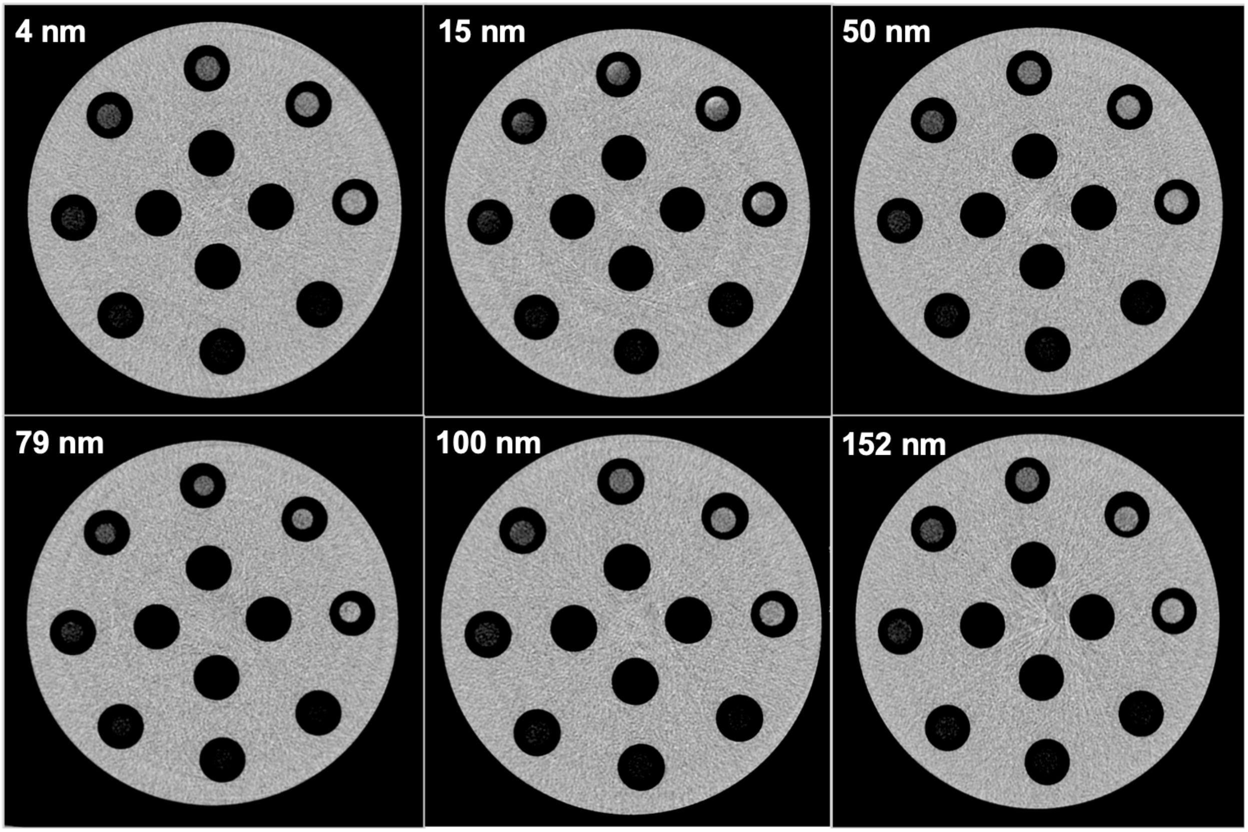

Supporting Figure 5: Conventional CT equivalent images of all AuNP sizes generated from SPCCT phantom scans. Images are displayed in window level of 250 HU and window width of 500 HU.

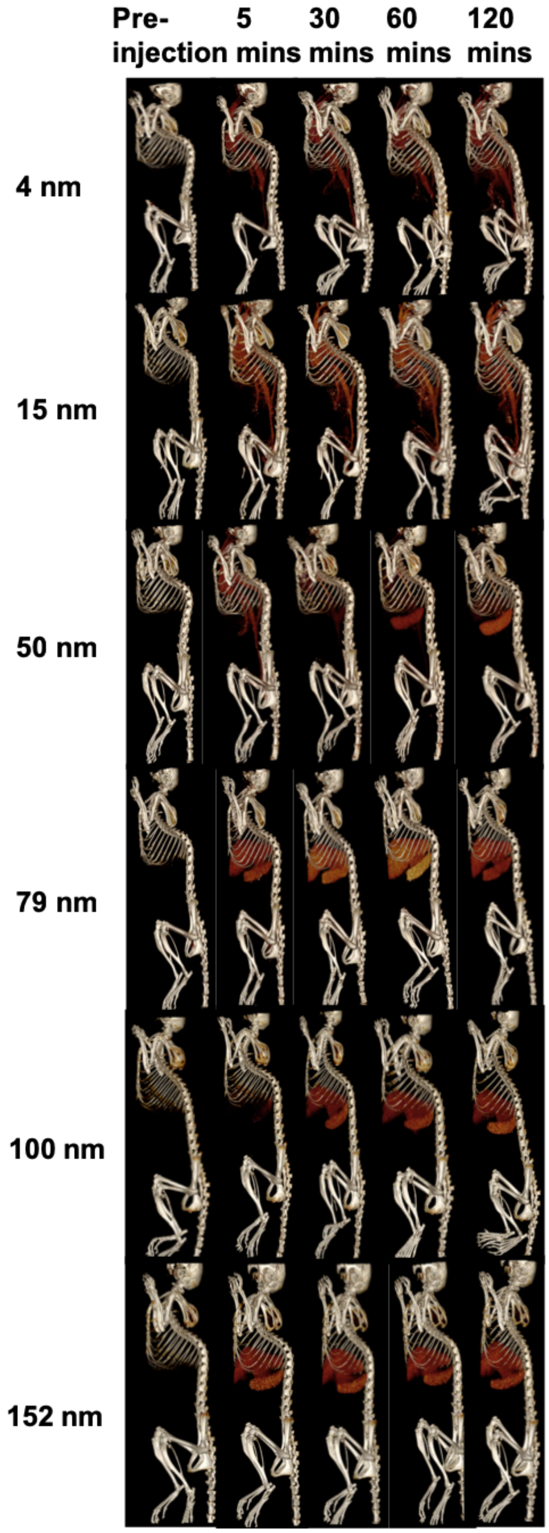

Supporting Figure 6: 3D volume rendered CT images at different post-injection time points. Images are displayed in window level of 1090 HU and window width of 930 HU.

		Intraclass Correlation	95% Confidence Interval	
			Lower Bound	Upper Bound
In vivo CT scan	Single Measures	.954	.947	.960
	Average Measures	.977	.973	.980
Phantom scan	Single Measures	.974	.968	.978
	Average Measures	.987	.984	.989

Supporting Table 1: Intraclass correlation coefficients calculated with a two-way mixed effect model for both Phantom scan CT contrast quantification and *in vivo* CT contrast quantification.

	p-value									
	5 minutes					30 minutes				
Comparison	B	L	S	K	T	B	L	S	K	T
4 nm vs. 15 nm	0.12	>0.99	0.99	>0.99	0.88	0.05	0.98	0.94	>0.99	0.72
4 nm vs. 50 nm	0.96	0.99	>0.99	0.97	0.83	>0.99	>0.99	0.95	>0.99	0.88
4 nm vs. 79 nm	0.39	0.20	0.77	0.99	0.85	<0.01	0.09	0.93	<0.01	0.19
4 nm vs. 100 nm	<0.01	<0.01	0.81	0.39	<0.01	<0.01	<0.01	0.69	<0.01	0.46
4 nm vs. 152 nm	<0.01	0.05	0.94	<0.01	0.53	<0.01	0.05	>0.99	<0.01	0.60
15 nm vs. 50 nm	0.40	0.91	>0.99	0.97	>0.99	<0.01	0.90	0.49	>0.99	>0.99
15 nm vs. 79 nm	<0.01	0.09	0.39	0.99	>0.99	<0.01	0.01	0.44	<0.01	0.92
15 nm vs. 100 nm	<0.01	<0.01	0.41	0.39	<0.01	<0.01	<0.01	0.17	<0.01	>0.99
15 nm vs. 152 nm	<0.01	0.02	0.66	<0.01	0.99	<0.01	<0.01	0.70	<0.01	>0.99
50 nm vs. 79 nm	0.04	0.54	0.71	>0.99	>0.99	<0.01	0.26	>0.99	0.03	0.84
50 nm vs. 100 nm	<0.01	0.01	0.75	0.85	<0.01	<0.01	<0.01	>0.99	<0.01	0.99
50 nm vs. 152 nm	<0.01	0.22	0.91	0.02	>0.99	<0.01	0.18	>0.99	0.01	>0.99
79 nm vs. 100 nm	0.12	0.60	>0.99	0.82	<0.01	0.93	0.21	>0.99	>0.99	>0.99
79 nm vs. 152 nm	0.01	>0.99	>0.99	0.02	>0.99	0.97	>0.99	>0.99	>0.99	0.98
100 nm vs. 152 nm	0.53	0.91	>0.99	0.28	<0.01	>0.99	0.31	0.96	>0.99	>0.99
	60 minutes					120 minutes				
Comparison	B	L	S	K	T	B	L	S	K	T
4 nm vs. 15 nm	0.24	0.94	0.94	>0.99	0.83	0.21	0.98	0.74	>0.99	0.97
4 nm vs. 50 nm	0.85	0.92	0.53	0.86	0.83	0.01	0.13	0.21	0.12	0.89
4 nm vs. 79 nm	<0.01	<0.01	0.27	0.01	0.43	<0.01	<0.01	0.73	<0.01	0.44
4 nm vs. 100 nm	<0.01	<0.01	0.56	<0.01	0.27	<0.01	<0.01	0.93	<0.01	0.24
4 nm vs. 152 nm	<0.01	0.05	>0.99	<0.01	0.92	<0.01	0.10	>0.99	<0.01	0.63
15 nm vs. 50 nm	<0.01	0.42	0.11	0.86	>0.99	<0.01	0.02	<0.01	0.12	>0.99
15 nm vs. 79 nm	<0.01	<0.01	0.04	0.01	0.98	<0.01	<0.01	0.08	<0.01	0.89
15 nm vs. 100 nm	<0.01	<0.01	0.11	<0.01	0.94	<0.01	<0.01	0.18	<0.01	0.72
15 nm vs. 152 nm	<0.01	<0.01	0.78	<0.01	>0.99	<0.01	0.02	0.89	<0.01	0.97
50 nm vs. 79 nm	<0.01	0.01	>0.99	0.31	0.99	0.55	0.61	0.96	0.92	0.98
50 nm vs. 100 nm	<0.01	<0.01	>0.99	0.03	0.96	0.15	0.15	0.75	0.29	0.91
50 nm vs. 152 nm	<0.01	0.42	0.83	0.09	>0.99	0.26	>0.99	0.16	0.40	>0.99
79 nm vs. 100 nm	0.97	>0.99	>0.99	0.94	>0.99	0.98	0.97	>0.99	0.90	>0.99
79 nm vs. 152 nm	0.99	0.65	0.57	>0.99	0.96	>0.99	0.68	0.62	0.94	>0.99
100 nm vs. 152 nm	>0.99	0.38	0.86	>0.99	0.90	>0.99	0.19	0.85	>0.99	>0.99

Supporting Table 2: P-values of every possible pairs of AuNP sizes in across all organs for attenuation changes over 2 hours imaging time points. The pairs that have statistically significant differences in change of attenuation are highlighted in blue. Organs names are abbreviated: B-Blood, L- Liver, S- Spleen, K- Kidney, T- Thigh.

	p-value				
**Comparison**	Blood	Liver	Lung	Spleen	Kidney
**4 nm vs. 15 nm**	0.71	0.99	>0.99	0.99	0.05
**4 nm vs. 50 nm**	0.46	0.03	0.99	<0.01	0.92
**4 nm vs. 79 nm**	0.44	0.02	0.58	<0.01	0.35
**4 nm vs. 100 nm**	>0.99	<0.01	0.03	<0.01	0.32
**4 nm vs. 152 nm**	0.99	0.15	<0.01	<0.01	0.08
**15 nm vs. 50 nm**	0.99	0.03	>0.99	<0.01	<0.01
**15 nm vs. 79 nm**	0.99	0.02	0.68	<0.01	<0.01
**15 nm vs. 100 nm**	0.75	<0.01	0.04	<0.01	<0.01
**15 nm vs. 152 nm**	0.53	0.13	<0.01	0.02	<0.01
**50 nm vs. 79 nm**	>0.99	0.99	0.84	0.99	0.91
**50 nm vs. 100 nm**	0.49	0.12	0.08	0.99	0.91
**50 nm vs. 152 nm**	0.30	0.95	<0.01	0.08	0.51
**79 nm vs. 100 nm**	0.47	0.33	0.61	>0.99	>0.99
**79 nm vs. 152 nm**	0.29	0.80	0.19	0.02	0.98
**100 nm vs. 152 nm**	0.99	0.01	0.98	0.02	0.97

Supporting Table 3: P-values of every possible pairs of AuNP sizes in across all organs for biodistribution at 2 hours post-injection. A Tukey's multiple comparisons test was used to compare the interactions between each AuNP size. The pairs that have statistically significant differences are highlighted in blue.
